# Blue-Violet Light Irradiation Dose Dependently Decreases Carotenoids in Human Skin, Which Indicates the Generation of Free Radicals

**DOI:** 10.1155/2015/579675

**Published:** 2015-02-09

**Authors:** Staffan Vandersee, Marc Beyer, Juergen Lademann, Maxim E. Darvin

**Affiliations:** Charité-Universitätsmedizin Berlin, Department of Dermatology, Venereology and Allergology, Charitéplatz 1, 10117 Berlin, Germany

## Abstract

In contrast to ultraviolet and infrared irradiation, which are known to facilitate cutaneous photoaging, immunosuppression, or tumour emergence due to formation of free radicals and reactive oxygen species, potentially similar effects of visible light on the human skin are still poorly characterized. Using a blue-violet light irradiation source and aiming to characterize its potential influence on the antioxidant status of the human skin, the cutaneous carotenoid concentration was measured noninvasively in nine healthy volunteers using resonance Raman spectroscopy following irradiation. The dose-dependent significant degradation of carotenoids was measured to be 13.5% and 21.2% directly after irradiation at 50 J/cm² and 100 J/cm² (*P* < 0.05). The irradiation intensity was 100 mW/cm². This is above natural conditions; the achieved doses, though, are acquirable under natural conditions. The corresponding restoration lasted 2 and 24 hours, respectively. The degradation of cutaneous carotenoids indirectly shows the amount of generated free radicals and especially reactive oxygen species in human skin. In all volunteers the cutaneous carotenoid concentration dropped down in a manner similar to that caused by the infrared or ultraviolet irradiations, leading to the conclusion that also blue-violet light at high doses could represent a comparably adverse factor for human skin.

## 1. Introduction

The exposure of the body to electromagnetic irradiation is a ubiquitous and lifelong event with numerous beneficial and adverse effects on the individual. Natural sunlight is by far the most important irradiation source. Visible (VIS) light (380–760 nm) represents about 40% of the emitted energy that reaches the surface of the earth [1, 2]. Nevertheless, most biological effects are attributed to the ultraviolet (UV) spectrum (290–400 nm), the rate of which is influenced by the latitude and the ozone layer's condition. The numerous effects of UV light on human skin have been subject to thorough research for decades [3]. Despite lacking the potential to penetrate deeply into the skin, UVB (290–320 nm) and UVA (320–400 nm) light exhibit a broad range of beneficial as well as adverse effects. Most of the latter effects are based on UV-induced DNA-mutation and immunosuppression [4], causing photoaging [5] and especially carcinogenesis [6]. Thus, the first sunscreens developed focused on blocking UVB though primarily for sunburn prevention. Then, after discovering its predominantly immunosuppressive effects, also the UVA range became interesting [7, 8]. Defective DNA or cell membranes, antioxidant depletion, proinflammatory effects, collagen, and elastin degradation represent the most important causes of UV-induced skin damage [9–11]. These effects are associated with the direct photochemical effects of UV and with indirect effects related to the action of free radicals and especially reactive oxygen species (ROS), generated subsequent to UV exposure [12–14].

With these effects well addressed, and potent means of protection being available, the assessment of infrared (760–3,000 nm) (IR) and VIS in the context of photodamage moved into focus. Since 1982 [15] scientific groups worldwide have been investigating the influence of IR on the skin showing its capability of ROS generation [16–19]. In 2006, the induction of ascorbate radicals by VIS could be shown* ex vivo* [20]. In 2008, Cho et al. proved that VIS and IR in conjunction induce skin damage* in vivo*, using the activation of matrix metalloproteinases (MMPs) and procollagen I formation as surrogate markers for the cutaneous degradation [21]. In 2009, Zastrow et al. [22] presented the action spectrum of the formation of free radicals in the skin* ex vivo* in the range between 280 and 1.600 nm. The free radicals were shown to be generated in the whole spectral range with the maximum in the UV. In 2010, the induction of ROS in the skin subsequent to IR-A (760–1.440 nm) and NIR (760–3.000 nm) irradiation was described [23, 24]. Another explanation for these findings was proposed showing the possible formation of heat-shock radicals in the skin subsequent to IR irradiation [25, 26]. However, in 2014, the different pathways for IR-induced ROS and heat-shock-induced ROS in the skin were characterized, postulating the generation of free radicals including ROS in the human skin subsequent to IR exposure independent of the applied doses [2]. In 2012, the production of ROS, proinflammatory cytokines, and MMP-1 expression in human skin subsequent to VIS exposure was reported [27]. Moreover, as the ROS generation is much greater in living skin in comparison to excised skin, the* in vivo* measurements are more informative than* ex vivo* measurements in this regard [28].

Blue-violet light (380–495 nm) is widely used in medicine for treatment of acne [29–31], psoriasis [32, 33], atopic dermatitis [34], and neonatal jaundice [35] and for wound healing [36, 37]. Moreover, the skin exposure to blue light results in antibacterial [38–40], antimicrobial [36, 41], and anti-inflammatory [42] effects. The side effects of blue-light therapy of neonates [43], suppression of dendritic cell activation [44] and effect on human dermal fibroblasts [45, 46] were also reported. The toxic effect of blue light on the skin was shown to be related to the generation of nonenzymatic nitric oxide (NO) radicals [47–49]. Blue-light-induced NO generation was proposed to be effective for therapy of local or systemic hemodynamic disorders [49]. The cytocidal effect of blue light was even proven to be effective for treatment of superficial skin carcinomas in humans [50]. Moreover, it could be speculated that blue-light-induced ROS are responsible for the antibacterial and antimicrobial effects mentioned above.

Several systems physiologically protect the skin from oxidative stress. In addition to enzymes such as catalase, glutathione peroxidase, or superoxide dismutase, this group comprises substances that cannot be synthesized by the human organism, such as vitamins (C and E), carotenoids, flavonoids, and phytoestrogens [51, 52]. The important protective role of carotenoids in neutralizing excessive free radicals could be shown* in vivo* [53–55] as could their potential to act as representative markers for the antioxidant status of the human epidermis [56, 57].

That latter feature makes them suitable for assessing the potential effects of VIS on the skin as well. In addition, carotenoids can be dietetically supplied [58] and have been described to contribute to an enhanced cutaneous UV protection [59, 60]. This and the fact that they seem to generally correlate with the individual stress level and lifestyle factors such as alcohol consumption, smoking, and current health stage have highlighted their importance in an even more general context [61–63].

Thus, in order to assess the dose-dependent influence that blue-violet light can potentially exert on the antioxidant status of the skin, we exposed nine healthy volunteers to different dosages of blue-violet light and measured the kinetics course of the cutaneous carotenoid concentration by means of resonance Raman spectroscopy over a period of 24 hours after irradiation.

## 2. Material and Methods

### 2.1. Volunteers

Nine healthy volunteers (see Table 1 for volunteers' characteristics) aged between 26 and 63 years with skin types II and III according to the Fitzpatrick classification [64] were included in the study. Exclusion criteria were any kind of cutaneous diseases, pregnancy, consumption of potentially phototoxic drugs of any kind, intensive sun exposure during the last 2 weeks, and hair existence on the inner forearm. The investigations were carried out in accordance with the ethical guidelines of the Declaration of Helsinki and had been approved by the Ethics Committee of the Charité-Universitätsmedizin Berlin before the study had started. All volunteers gave their informed written consent.

The volunteers were instructed not to utilize any skin care products on the forearm for at least 72 hours and not to bathe or shower for at least 4 hours prior to the beginning and during the experiments.

### 2.2. Source of Blue-Light Irradiation

**Figure 1:**
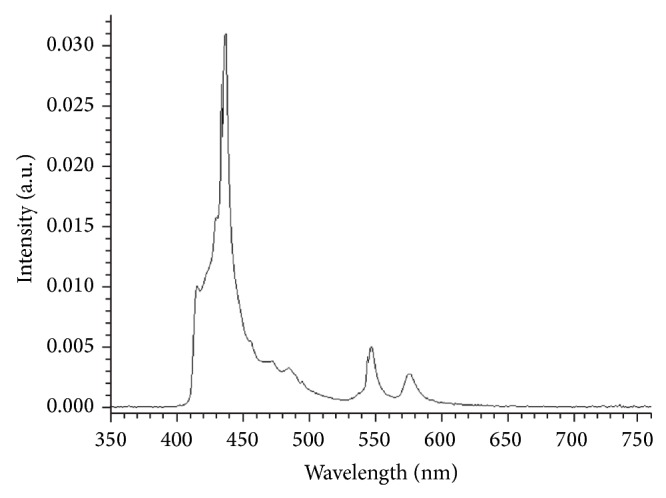
The emission spectrum of blue-light source of irradiation.

The skin was irradiated with blue-violet light (80% in the range 380–495 nm, maxima at 440 nm), which is normally used for defining the skin treatment area for digital phototherapy using a skintrek PT3, Lumedtec GmbH, Lüneburg, Germany. The emission spectrum can be seen in Figure 1.

### 2.3. Resonance Raman Spectroscopy

The carotenoids were measured in the volunteers' skin noninvasively using resonance Raman spectroscopy [65, 66]. Based on the absorption properties of the carotenoids, the resonant excitation is achieved in the blue range of the spectra [66]. Thus, the 488 nm blue-light wavelength of an argon CW laser was used as a source of carotenoid excitation. Under the applied excitation conditions, the carotenoid Raman lines are resonantly enhanced and clearly detected in the fingerprint range of the spectra on the high fluorescence background. The intensity of the carbon-carbon double-bond stretch vibration of the conjugated backbone of carotenoid molecules (C=C) measured at 1525 cm^−1^ was analyzed. This was done due to the fact that the C=C chemical bonds of the carotenoid molecules are responsible for their antioxidant properties when the free radicals are neutralized [60, 67, 68]. Thus, the reaction with free radicals induces the destruction of the carotenoids' C=C bonds that is attributed to the decrease of the 1525 cm^−1^ Raman peak intensity. Our group has previously described the Raman device utilized in the present study in detail [66]. Resonance Raman spectroscopy was chosen in consideration of the strong advantages over other noninvasive methods [69].

### 2.4. Temperature Measurement

The skin surface temperature was measured using a noncontact thermometer (Rytek Schlender Messtechnik, Rüthnick, Germany).

### 2.5. Measurement of Light Intensity

The intensity of blue light was measured with a power meter (Hydrosun Medizintechnik GmbH, Type HBM-1, Müllheim, Germany).

### 2.6. Study Design

Two areas of 2 × 2 cm^2^ were marked on the left and right inner forearms of the volunteers. Afterwards, the carotenoid concentration was measured five times at different places within the marked areas and the mean values were calculated as initial concentration. Then the blue-violet light irradiation was conducted by administering 50 J/cm^2^ on the one forearm first and subsequently 100 J/cm^2^ on the other forearm. The left or right forearms were randomized from volunteer to volunteer. To achieve the intended doses, the light source was positioned at such a distance to the skin surface that the light intensity exactly amounted to 100 mW/cm^2^ (individually controlled by means of a power meter). Thus, the irradiation time was calculated to be 500 sec. and 1000 sec. in order to achieve a dose of 50 J/cm^2^ and 100 J/cm^2^, respectively. Note that this is far above the intensity of illumination present, when using the device for UV-therapy planimetry in the therapeutical setting, which is about 3.7 mW/cm^2^ (dose does not exceed 0.25 J/cm^2^). The carotenoid concentration was then measured directly, as well as 1, 2, and 24 hours after blue-violet light irradiation. To exclude the formation of heat-shock radicals, the skin was continuously cooled during the irradiation by a fan and the skin temperature remotely measured with a thermometer before, during, and after irradiation.

The applied irradiation doses were selected as they represent a dosage that is readily acquirable under natural conditions by sunbathing. The range between 380 and 495 nm (violet and blue range of the spectra) represents 80% of the energy emitted by the skintrek illumination source (see Figure 1). In this wavelength range, according to the currently active ASTM sun reference spectrum, the emitted average energy accounts for about 161 W/m^2^ in the USA [70]. This value corresponds to sun exposure in southern Europe. This means that the acquired dosage after one hour of sunbathing at noon would amount to about 57 J/cm^2^, which is in the range of our experimental settings (50 J/cm^2^ and 100 J/cm^2^).

### 2.7. Statistical Analyses

Statistical analyses were done by one-way or two-way analysis of variance (ANOVA) as appropriate. All analyses were conducted with GraphPad Prism, 4.0 (GraphPad Software Inc., La Jolla). Differences were considered to be significant at *P* < 0.05.

## 3. Results and Discussion

**Figure 2:**
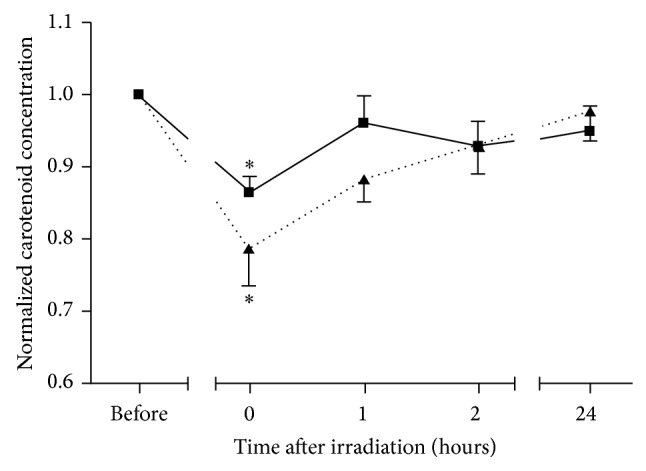
The kinetic course of the cutaneous carotenoids degradation after the irradiation with blue light at doses of 50 J/cm^2^ (mean + SD, solid line) and 100 J/cm^2^ (mean − SD, dotted line) and the subsequent restoration. ∗ shows the significant degradation in comparison to the initial level before irradiation (*P* < 0.05).

Figure 2 displays the kinetic course of the cutaneous carotenoids degradation after the irradiation with blue-violet light at doses of 50 J/cm^2^ and 100 J/cm^2^ and the subsequent restoration. A statistically significant decrease of the carotenoid concentration after the application of both doses was observed. The obtained mean magnitude of carotenoid destruction was determined as the difference between the carotenoid values measured before and directly after blue-violet light irradiation. It is about twice as high at a dosage of 100 J/cm^2^ compared to 50 J/cm^2^ (21.2% versus 13.5%). The median skin surface temperature measured immediately after irradiation was 36.3°C (range 34.9–37.8) for 50 J/cm^2^ and 36.1°C (range 32.2–38.5) for 100 J/cm^2^. The initial skin temperature measured before irradiation was 31.5°C on average. No volunteer uttered any inconvenient sensations or other problems during the entire experiment. Some volunteers reported they felt only a slight skin temperature increase when the respective skin areas were exposed to the blue-violet light.

The carotenoid degradation in human skin subsequent to irradiation with blue-violet light can be explained by, inter alia, the direct carotenoid destruction by blue-violet light absorption, the effect of heat-shock radicals generated due to the temperature increase, and the generation of free radicals including ROS due to the blue-violet light activation of mitochondrial activity. The two first possible reasons can be eliminated as carotenoids are very stable under the applied irradiation conditions (the dissociation energy of carotenoid molecules is higher than the applied irradiation energies) [71, 72]. The temperature starts to play an important role in the generation of heat-shock radicals in the skin only when exceeding the value of 39°C [2]. The obtained reduction of the cutaneous carotenoid concentration subsequent to irradiation of human skin with blue-violet light is fully in correlation with the results obtained for UV [73], IRA, and NIR [74] irradiation of human skin* in vivo*, which were associated with the generation of free radicals and especially ROS in the skin. Thus, the dose-dependent generation of blue-violet light-induced ROS in the skin can be established confirming the results obtained previously [22, 49].

When being irradiated at doses of 50 J/cm^2^, the carotenoid concentration in the skin was already restored one hour after exposure. In contrast to this, the restoration of the carotenoid concentration to the initial level takes 24 hours after irradiation when being irradiated at doses of 100 J/cm^2^. Still, 2 hours after irradiation at 100 J/cm^2^, almost 50% of the degraded carotenoids were restored. The restoration of cutaneous carotenoids after the irradiation with blue-violet light shows dose-dependent kinetics. Higher irradiation doses give rise to the generation of a higher amount of ROS and, therefore, to the depletion of higher amount of carotenoids. Consequently, more time is required to restore the carotenoid level that existed prior to irradiation. Conversely, low irradiation doses destroy a lower amount of carotenoids, which reduces the time span for restoration. The restoration efficacy is expected to be influenced by carotenoid-rich nutrition that was shown in other studies [75–77].

To determine whether or not the reference blue excitation light used in the applied resonance Raman system may trigger the same effects in the skin as irradiation with blue-violet light, the applied irradiation doses should be compared. The power of the utilized reference excitation blue light was 10 mW. The diameter of the excitation beam was 6.5 mm in order to eliminate the influence of skin inhomogeneities on the Raman measurements [65]. The exposure time was 5 sec. Thus, the illumination dose of the reference blue light was equal to 0.15 J/cm^2^ and this is substantially lower than the applied blue-violet light irradiation doses (0.15 J/cm^2^ versus 50/100 J/cm^2^). Therefore, no detectable effect of any reference excitation blue light on human skin can be affirmed.

While in the past sunscreen research addressed the measuring technology in order to exactly specify not only the UVB but also the UVA spectral ranges as a prerequisite for developing new or improving existing products, Zastrow et al. [22] revealed in 2009 that approximately 50% of the free radicals, which are induced by solar radiation in the human skin, originate from visible and infrared light. Although the radical formation in the infrared spectral range has become a topic of intense international research, studies demonstrating that high amounts of free radicals are also induced by visible and infrared light are rare. The presented study well demonstrates that visible blue-violet light also induces free radicals in human skin* in vivo*. The generation of free radicals in the remaining visible spectrum is highly probable and, therefore, is expected. These findings have the potential to provide fresh impetus to the development of sunscreens, in particular with regard to transferring from UV protection to protection in the whole solar spectrum.

## 4. Conclusions

The irradiation of the human skin with blue-violet light results in a dose-dependent significant degradation of the epidermal antioxidants (*P* < 0.05) as was shown by* in vivo* measurements of the carotenoid concentration using resonance Raman spectroscopy. The mean magnitude of the carotenoid destruction was determined to be 13.5% after irradiation at 50 J/cm^2^ and 21.2% after irradiation at 100 J/cm^2^. Depending on the irradiation dose, the restoration time was measured to be 1 hour for the dose of 50 J/cm^2^ and 24 hours for the dose of 100 J/cm^2^. The increase of the irradiation dose is likely to extend also both the magnitude of carotenoid destruction and the restoration time.

Based on the results obtained previously by other scientific groups, it could be established that free radicals and most probably ROS are generated in the human skin subsequent to irradiation with blue-violet light* in vivo*.

The obtained results could be essential for the development of future protection strategies based not only on UV but also on protection across the whole spectrum of the light.

## Figures and Tables

**Table 1:** Volunteer's characteristics.

Parameters	Mean (range)
Number of volunteers	9
Age	39.8 (26–63)
Gender	7 females, 2 males
Skin type according to Fitzpatrick	Type II: 8, type III: 1
Smoker/nonsmoker	2/7
Vegetarian/nonvegetarian	1/8
